# Advancements of Image Processing and Vision in Healthcare

**DOI:** 10.1155/2018/8458024

**Published:** 2018-01-15

**Authors:** Md Atiqur Rahman Ahad, Syoji Kobashi, João Manuel R. S. Tavares

**Affiliations:** ^1^Department of Electrical and Electronic Engineering, University of Dhaka, Dhaka, Bangladesh; ^2^Graduate School of Engineering, University of Hyogo, Himeji, Japan; ^3^Instituto de Ciência e Inovação em Engenharia Mecânica e Engenharia Industrial, Departamento de Engenharia Mecânica, Faculdade de Engenharia, Universidade do Porto, Porto, Portugal

## 1. Introduction

Advancements of image processing and computer vision in healthcare sectors are required to be explored. Though this arena has been expanded a lot in the last few decades, still the progresses are not satisfactory. Hence, we have endeavored to delve into the healthcare using image processing and computer vision, though sensor-based activity is also a very important area [1, 2]. Integration of various cues and modalities can enhance the performance of image- or vision-based analysis. This special issue demanded to cover the broad spectrum that benefits from the automatic understanding of medical healthcare image analysis and related topics. This special issue accepted high-quality research papers as well as review articles covering both the challenges and applications of image processing and vision in healthcare, for the betterment of human life.

Various important areas are covered here, broadly medical imaging for healthcare, vision systems in healthcare applications, pattern recognition related to healthcare, big data and data mining in healthcare, multimodal integration for healthcare, affective computing, biometrics issues related to healthcare, and action or emotion or behavior analysis/recognition [2, 3] for healthcare applications, and so forth. For example, in order to develop smart homes for elderly people, comprehending various activities based on image or video or sensor are very crucial. Though we concentrated mainly on video-based analysis from a digital camera or similar image sensors, normal and abnormal daily activity understandings/recognition [4] based on other sensors are widely explored in the last several years. This special issue was approachable in other related areas too, for example, related databases, special system or instrumentations related to healthcare, nurse robot, big data, assistive technologies, and applications.

## 2. Various Approaches

We have received thirty-two manuscripts for this special issue under the umbrella of various advancements of imaging and vision in healthcare. Core topics of the submissions are brain detection, prediction in hypertension-nondiabetic patients, patient position on the goniometric assessment task, deconvolution model for MTF improvement in CT, knee arthroplasty, radiomics for cerebral aneurysm occurrence in MR angiography images, OCT retinal image segmentation, knee joint kinematics recognition, shape change analysis of human brain, classification of microscopic colonic images, 3D macrophage tracking, deep neural network-based medical image compression, reconstruction quality in digital breast tomosynthesis, phase-contrast X-ray imaging, medical image restoration, brain tumor growth investigation, eyepointer interaction device, brain abnormality detection, quality index of medical images, brain MRI segmentation, assessment of bulged disk cervical vertebral, blue-white structure detection in dermoscopy images, and so forth.

Among 32 different submissions from various countries, we finally accepted 9 quality papers. None of these papers are from guest editors. For the final acceptance of these papers, the average processing period was 4.5 months, after 2 or 3 revisions.

Y. Huang et al. presented intracellular mobility based on adaptive total variation optical flow model. They extracted the histograms of oriented optical flow (HOOF) [5] after the optical flow of intracellular mobility was calculated. Then, distances of different HOOFs were computed as the motion features. These are covered in “Quantitative Analysis of Intracellular Motility Based on Optical Flow Model.”

S. Han et al. proposed a deconvolution-based model for modulation transfer function (MTF) improvement in CT to achieve uniform spatial resolution. They considered eleven subband regions that can reduce noise as well as enhance spatial resolution. By exploiting approximate blurring point spread function (PSF) kernel, they proposed the work as “A Subband-Specific Deconvolution Model for MTF Improvement in CT.” It can perform well even in the soft tissue region.

The paper by V. Kelkar et al. shows variants of histogram shift method to enhance the hiding capacity for telemedicine application. In their reversible watermarking method for medical images, they achieved high peak signal-to-noise ratio (PSNR). The higher the PSNR value, the better the imperceptibility of the watermarking. They demonstrated better performance than classical histogram shifting-based algorithms [6] for this purpose.

Y.-M. Chen and S.-G. Miaou offered a new idea for noninvasive anemia detection in their paper titled “A Kalman Filtering and Nonlinear Penalty Regression Approach for Noninvasive Anemia Detection with Palpebral Conjunctiva Images.” In their anemia examining method, they considered a modified Kalman filter [7] along with a regression method with a penalty function. Their results were good compared with other similar methods.

V. Roy et al. proposed a method to remove motion artifacts from EEG signals. In the paper “Gaussian Elimination-Based Novel Canonical Correlation Analysis method for EEG Motion Artifact Removal,” they improved the canonical correlation analysis (CCA) [8] based approach by introducing Gaussian elimination method, called GECCA. Their approach is found to be better than similar few other CCA-based methods for removing EEG motion artifacts.

The paper by L. Fassina et al. evaluated the dynamics and kinematics of beating cardiac syncytia and studied the inotropic effects of electromagnetic simulation and so forth. In their work “Model of Murine Ventricular Cardiac Tissue for *In Vitro* Kinematic-Dynamic Studies of Electromagnetic and *β*-Adrenergic Stimulation,” they designed an *in vitro* model of murine ventricular cardiac tissue so that they can study various related parameters, for example, the contraction movement.

In this special issue, we could accept a few excellent review papers as well. Augmented reality (AR) becomes very crucial in various vision-based applications, including medical surgery. In this special issue, one of the papers, written by P. Vávra et al. under the title of “Recent Development of Augmented Reality in Surgery: A Review,” demonstrated the reality of the augmented reality in surgical procedures. In this paper, we can get an in-depth summary and evaluation of recent works (from 2010 till 2016), published in PubMed and SCOPUS on AR and surgery. From several hundred research papers, they finally covered about one hundred important and related works to demonstrate the state of the art on AR-based surgery. Various application areas and future challenges are also profoundly addressed in this work.

Another review work is also published in this volume by R. Richert et al. In the paper “Intraoral Scanner Technologies: A Review to Make a Successful Impression,” R. Richert et al. reviewed intraoral scanner (IOS) technologies for dental and clinical applications. They highlighted the current technologies on IOS and their respective clinical approaches and impacts. Finally, they addressed a very important aspect, that is, the accuracy of IOS technologies, precision and trueness of IOS files, and the intermaxillary relationship to perform prosthetic rehabilitation for patients.

M. Machoy et al. comprehensively presented a review of the applications of optical coherence tomography (OCT) in dental diagnostics. At the basic level, various types of OCT and its operating principle are addressed. OCT can be used in various applications, but this survey addressed the involvement of OCT in the arena of dentistry. A number of tables illustrated the OCT facilities, OCT in cardiology and restorative dentistry, in endodontics, in prosthetics, in diagnostics of oral tissues and implantology, in orthodontics, and so on in the last five years. These can be very useful for researchers.

## 3. Conclusion

In this short note, we presented the background and papers that are covered in the special issue on the advancements of image processing and computer vision for healthcare. From 32 submissions, 9 papers are finally selected with the rigorous review process. These works will contribute to the community. We wish to have more research works on healthcare.
